# Efficacy and safety of PD-1/PD-L1 inhibitors versus chemotherapy or cetuximab in the treatment of platinum-refractory recurrent or metastatic head and neck squamous cell carcinoma: a meta-analysis

**DOI:** 10.3389/fphar.2026.1675407

**Published:** 2026-07-31

**Authors:** Zhongji Chen, Zhong Zhong, Weiming Liang, Qiwen Feng, Minyao Wu, Lidai Lin, Guanlan Huang, Xiaoxia Tang, Jieru Quan, Jiayong Wei, Jiongying Qin

**Affiliations:** 1 The First Affiliated Hospital of Guangxi University of Science and Technology, Guangxi University of Science and Technology, Liuzhou, China; 2 Department of Otorhinolaryngology and Head and Neck Surgery, Nanning First People’s Hospital (The Fifth Affiliated Hospital of Guangxi Medical University), Nanning, China; 3 School of Economics and Management, Guangxi University of Science and Technology, Liuzhou, China

**Keywords:** meta-analysis, metastatic, nasopharynx, overall survival, PD-1 inhibitor, PD-L1 inhibitor, platinum-refractory, squamous cell carcinoma of head and neck

## Abstract

**Introduction:**

This meta-analysis was designed to compare the efficacy and safety of PD-1/PD-L1 inhibitors versus chemotherapy or cetuximab in the treatment of platinum-refractory recurrent/metastatic head and neck squamous cell carcinoma (R/M HNSCC).

**Materials and Methods:**

Four databases (PubMed, Embase, Web of Science, and Cochrane Library) were searched for RCTs comparing PD-1/PD-L1 inhibitors versus chemotherapy or cetuximab in the treatment of platinum-refractory R/M HNSCC, from the database’s establishment to 2 January 2026. Progression-free survival (PFS), overall survival (OS), objective response rate (ORR), and treatment-related adverse events (trAEs) were subjected to meta-analyses.

**Results:**

Five RCTs were included in the meta-analysis. The meta-analysis included a group of 1,700 patients diagnosed with platinum-refractory R/M HNSCC. Within this cohort, 926 patients were administered PD-1/PD-L1 inhibitors, while 774 patients received chemotherapy or cetuximab. Compared with chemotherapy or cetuximab, PD-1/PD-L1 inhibitors yielded superior OS (HR: 0.80, 95% CI: 0.72 to 0.89, P < 0.01), lower incidence of grade 3–5 trAEs (RR: 0.37, 95% CI: 0.30 to 0.45, P < 0.01) and any grade trAEs (RR = 0.74, 95% CI: 0.69 to 0.79, P < 0.01). There was no statistically significant difference in the ORR (RR: 1.01, 95% CI: 0.71 to 1.51, P = 0.94) or PFS (HR: 1.00, 95% CI: 0.90 to 1.11, P = 0.99) between the two groups.

**Conclusion:**

The results indicated that PD-1/PD-L1 inhibitors were effective for platinum-refractory R/M HNSCC particularly in populations with high PD-L1 expression and exhibited a superior safety profile compared to chemotherapy or cetuximab. Additional well designed RCTs are required in the future to validate our conclusion.

**Clinical Trial Registration:**

https://www.crd.york.ac.uk/PROSPERO/view/CRD420251020053, identifier PROSPERO (CRD420251020053).

## 1 Introduction

Head and neck cancer ranks as the sixth most common malignancy globally, with over 891,000 new cases and more than 458,000 deaths annually, predominantly due to head and neck squamous cell carcinoma (HNSCC) (Bray et al., 2024). Nasopharyngeal carcinoma (NPC) is a form of head and neck cancer that usually develops around the ostium of the eustachian tube in the lateral wall of the nasopharynx (Chou et al., 2008). A class of solid tumors with a variety of biological characteristics, head and neck carcinomas include tumors of the larynx, oropharynx, oral cavity, hypopharynx, and nasopharynx (Adelstein et al., 2017; Marur and Forastiere, 2016). The most prevalent malignant tumor of the head and neck is called head and neck squamous cell carcinoma (Cabezas-Camarero and Pérez-Segura, 2022; Sung et al., 2021), Squamous cell carcinoma accounts for more than 90% of head and neck cancers (Pfister et al., 2020).

Patients with R/M HNSCC are classified as platinum-refractory R/M HNSCC if the cancer relapses or spreads within three to 6 months of stopping platinum-containing chemotherapy, or if the illness advances within 6 months of starting platinum-based treatment (Bernier, 2008; Saloura et al., 2014), They have a median overall survival of no more than 6 months, a poor long-term prognosis, and few available treatment choices. Due to, treatment options for Platinum-refractory R/M HNSCC were limited to chemotherapy (including paclitaxel, docetaxel, or methotrexate) or cetuximab, which yield a median overall survival of 7 months or less, (Nccn, 2018; Licitra and Felip, 2009; Guardiola et al., 2004; Vermorken et al., 2008a; Tian et al., 2022), and median PFS of 2–3 months, and without clear demonstration of an improvement in OS (SEER, 2022a; SEER, 2022b; León et al., 2005; Fakhry et al., 2008), It further indicates the need to find better treatment options for patients with Platinum-refractory R/M HNSCC.

Before approvals of PD-1/PD-L1 inhibitors therapy (Administration UFaD, 2022; Agency, 2021; Ferris et al., 2018), EXTREME Program Platinum-based combination chemotherapy regimens and cetuximab are commonly used in the first-line recurrent and metastatic settings (Licitra and Felip, 2009), which provides median OS of about 10 months and is associated with substantial toxicity (Vermorken et al., 2008b). Despite a median overall survival exceeding 10 months, 82 percent of patients encountered grade 3–5 trAEs, and 20 percent ceased therapy due to AEs, underscoring the necessity for better tolerated and more efficacious therapeutic alternatives. Despite robust first-line treatment, certain patients may demonstrate resistance to platinum therapy, leading to disease progression. Therefore, it is essential to improve survival rates in platinum-refractory R/M HNSCC without utilizing platinum therapy. PD-1/PD-L1 inhibitors is starting to fulfill this unmet requirement (Galvis et al., 2020).

PD-1 is an important immune checkpoint molecule, PD-L1 is one of the ligands of PD-1 (Ai et al., 2020; Dermani et al., 2019; Chen and Mellman, 2017), which can enhance the immune response to tumors and impairs the growth of cancer cells (Asaoka et al., 2015). Blocking this pathway with antibodies against PD-1 or its ligand has produced significant clinical responses in many patients with different types of cancers, including melanoma, non-small cell lung cancer, renal cell carcinoma, bladder cancer and Hodgkin’s lymphoma (Ansell et al., 2015; Hamid et al., 2013; Herbst et al., 2014; Powles et al., 2014; Topalian et al., 2014; Brahmer et al., 2012; Topalian et al., 2012). Therefore, PD-1/PD-L1 inhibitors may help reduce the immunosuppression of tumors (Brahmer et al., 2012). In recent years, results indicate that pembrolizumab exhibited enhanced overall survival relative to cetuximab or chemotherapy, demonstrating non-inferiority in the total patient cohort (8.4 vs 6.9 months, hazard ratio 0.80, 0.65 to 0.98; nominal p = 0.0161). Four patients reported grade 3 or greater severe treatment-related adverse events (33 [13%] of 246 patients versus 85 [36%] of 234 patients) (Cohen et al., 2019a). The application of these immunotherapies led to a median OS ranging from 7.5 to 8.4 months. Currently, they are recommended as second-line options for patients with recurrent/metastatic head and neck squamous cell carcinoma (Ferris et al., 2018; Cohen et al., 2019b; Burtness et al., 2019). PD1/PD-L1 inhibitors, namely, pembrolizumab, nivolumab, and durvalumab, have obtained approval from the US. FDA for the treatment of platinum-refractory R/M HNSCC (Cohen et al., 2019b; NCCN, 2013; Farkona et al., 2016; Ferris et al., 2020). Highlighting the clinical value of PD-1/PD-L1 inhibitors in the context of HNSCC (Rischin et al., 2019).

This meta-analysis was to designed to compare the efficacy and safety of PD-1/PD-L1 inhibitors versus chemotherapy or cetuximab in the treatment of platinum-refractory R/M HNSCC.

## 2 Materials and methods

### 2.1 Search strategy

In compliance with the 2020 guidelines of the Preferred Reporting Project for Systematic Review and Meta-Analysis (PRISMA), the current meta-analysis was conducted. The present work has been officially recorded at PROSPERO under the registration number CRD420251020053. A systematic search was conducted in four databases, namely, PubMed, Embase, Web of Science, and the Cochrane Library, to identify literature published up to 2 January2026. The search strategy used a combination of MeSH and free-text words following the PICOS principle. The search keyword was “PD-1/PD-L1 inhibitor” AND “squamous cell carcinoma of head and neck” AND “platinum-refractory” AND “recurrent/metastatic”.

Supplementary Material 1 provided a comprehensive listing of the search results.

### 2.2 Inclusion and exclusion criteria

The criteria for inclusion were as follows (Bray et al., 2024): patients with platinum-refractory recurrent or metastatic head and neck squamous cell carcinoma (Chou et al., 2008); patients in the intervention group received PD1/PD-L1inhibitors (Adelstein et al., 2017); patients in the controlled group received chemotherapy or cetuximab (Marur and Forastiere, 2016); at least one of the following outcomes was reported: overall survival (OS), progression-free survival (PFS), ORR, and treatment-related adverse events (trAEs) (Cabezas-Camarero and Pérez-Segura, 2022). Study types: randomised controlled trials (RCTs).

The criteria for exclusion were as follows (Bray et al., 2024): Different types of papers, such as case reports, publications, letters, comments, reviews, meta-analyses, editorials, animal studies, protocols, conference, etc., (Chou et al., 2008); Other types of malignancies or diseases (Adelstein et al., 2017); Not relevant (Marur and Forastiere, 2016); Full text not available (Cabezas-Camarero and Pérez-Segura, 2022); Duplicate patient cohort (Sung et al., 2021); Data cannot be obtained.

### 2.3 Selection of studies

Selection of studies, including elimination of duplicates, was undertaken using EndNote (Version 20; Clarivate Analytics). An initial search was undertaken by two reviewers who independently deleted duplicate entries, assessed the titles and abstracts for relevance, and classified each study as either included or excluded. The settlement was arrived at through the attainment of consensus. A third reviewer would take on the role of an arbitrator if lacking a consensus.

### 2.4 Data extraction

Two independent researchers performed a meticulous analysis of the title and abstract, followed by a detailed perusal of the whole text. To address the inconsistencies, a third investigator was consulted. The data collected includes the name of the first author, The trial name, publication year, study design, number of patients, participant age, PD‐1/PD‐L1 inhibitors agent used, dosing schedule, ORR, PFS, OS, grade 3–5 trAEs and any grade trAEs. Reporting in the articles and Supplementary Material were obtained from each included RCTs.

Tumor PD-L1 expression could be measured using the Combined Positive Score (CPS) or the Tumor Proportion Score (TPS). CPS is calculated as the number of PD-L1-positive cells (including tumor cells, lymphocytes, and macrophages) divided by the total number of viable tumor cells, multiplied by 100. Assessment requires the presence of at least 100 viable tumor cells. TPS is defined as the percentage of tumor cells exhibiting membranous PD-L1 staining. In this meta-analysis, PD-L1 CPS ≥1 or PD-L1 TPS ≥1% was classified as PD-L1 expression-positive, whereas PD-L1 CPS <1 or the PD-L1 TPS <1% was defined as PD-L1 expression-negative.

### 2.5 Risk of bias assessment

Using the Cochrane Collaboration risk of bias assessment tool, two writers independently evaluated the methodological quality of each individual RCTs (Higgins et al., 2011). This RCTs assessed six key aspects: randomization, allocation concealment, blinding procedures, attrition rate, result reporting, and other sources of bias. The quality assessment results determined the labeling of each feature as low, unclear, or high risk. The quality assessment was conducted by two writers, and any discrepancies were resolved through consultation with a third reviewer. RevMan version 5.3 (Review Manager, The Cochrane Collaboration, 2020) was used to graphically depict the summary of bias risk.

### 2.6 Statistical analysis

The selection duplicate removal of studies included was conducted using EndNote (Version 20; Clarivate Analytics). The statistical analysis was performed using the Stata/SE version 12.0. The fixed-effects model incorporates the Relative Risk (RR) for Binary categorical outcomes in its initial structure. These metrics were reported together with their corresponding 95% confidence intervals (CI). The choice between fixed - effect and random - effect models was based on the I^2^ value and chi-square test P value. When heterogeneity was high (I^2^ >50%), the random - effect model was used. When heterogeneity was low (I^2^ ≤ 50%), the fixed - effect model was applicable. Statistical significance is defined as a p-value less than 0.05. Stata/SE version 12.0 was used to graphically depict the a overall hazard ratios (HR) for PFS and OS, we used funnel plot to evaluate the publication bias. Ultimately, a sensitivity analysis was conducted to assess the influence of different research on the combined findings and to evaluate the dependability of the results.

## 3 Results

### 3.1 Search results

**FIGURE 1:**
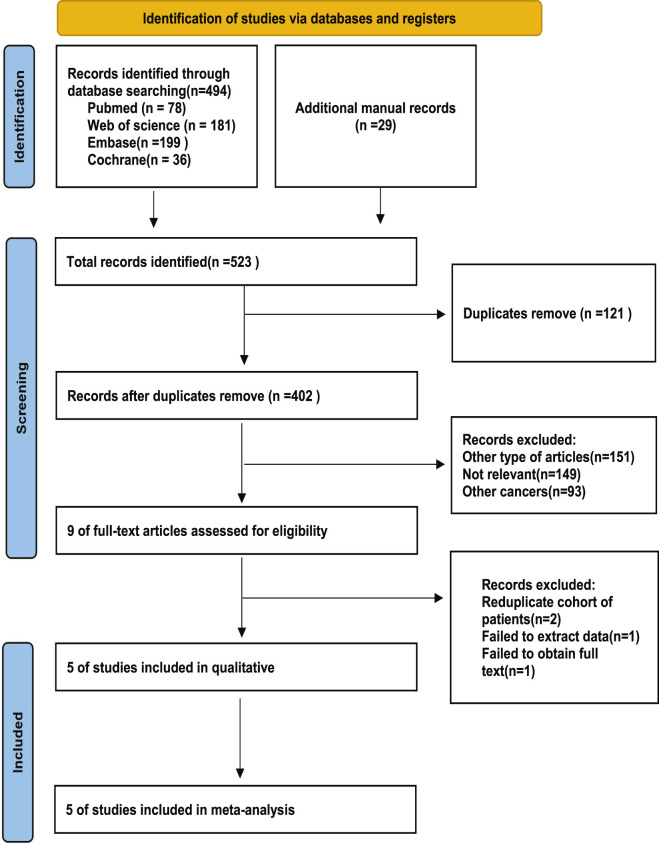
PRISMA flow diagram of the study selection process.

The procedure of selecting and integrating literature was depicted in Figure 1. A total of 523 records were initially identified. Following the removal of repeated records, a grand total of 402 records remained. Based on the evaluation of the titles and abstracts, a total of 397 publications were considered unsuitable and so eliminated. A comprehensive review of the whole text led to the selection of 5 RCTs finally included in this meta-analysis (Figure 1).

### 3.2 Patient characteristics

This meta-analysis included 5 RCTs (Ferris et al., 2018; Cohen et al., 2019a; Ferris et al., 2020; Even et al., 2021; Chan et al., 2023) published between 2018 and 2026. The studies encompassed 1,700 patients diagnosed with platinum-refractory R/M HNSCC. Of them, 926 patients were treated with PD1/PD-L1 inhibitors, whereas 774 patients were given chemotherapy or cetuximab. The trial identifier, authors, year, subgroup, sample, regimens, site of primary tumor, mean age (year), PD-L1 expression, race, ECOG PS(%) and tobacco use of the included literature were shown in Table 1. Among all the included randomized controlled trials, three articles did not restrict the primary tumor site (head and neck squamous cell carcinoma) (Ferris et al., 2018; Cohen et al., 2019a; Ferris et al., 2020), whereas the remaining two articles limited inclusion to tumors originating specifically from the nasopharynx (Even et al., 2021; Chan et al., 2023). Regarding the types of PD-1/PD-L1 inhibitors used, four articles reported employing PD-1 inhibitors (Pembrolizumab, Nivolumab, Spartalizumab) (Ferris et al., 2018; Cohen et al., 2019a; Even et al., 2021; Chan et al., 2023), and one article utilized a PD-L1 inhibitor (Durvalumab) (Ferris et al., 2020).

**TABLE 1:** Patient characteristics of included studies and patients.

Trial identifier	Author, year	Subgroup	Sample	Regimens	Site of primary tumor	Mean age (year)	PD-L1 expression		Race				ECOG PS (%)			Tobacco use	
							High	Low	White	Asian	Black	Other	0	1	2	Current/former	Never
KEYNOTE-040 (NCT: 02252042)	Cohen 2019 (36)	A	247	Pembrolizumab 200 mg Q3W	Oropharynx, hypopharynx	60 (55–66)	196	50	220	-	-	27	71	176	0	179	68
		B	248	Methotrexate, docetaxel, or cetuximab		60 (54–66)	191	54	218	-	-	30	67	180	1	182	66
CheckMate 141 (NCT: 02252042)	Ferris 2018 (21)	A	240	Nivolumab 3 mg/kg Q2W	Oral cavity, opharynx, hypopharynx	62 (23–91)	96	76	196	29	10	5	49	189	1	191	39
		B	121	Methotrexate, docetaxel, or cetuximab		63 (29–88)	63	40	104	14	3	0	23	94	3	85	31
KEYNOTE-122 (NCT: 02611960)	Chan 2023 (45)	A	117	Pembrolizumab 200 mg Q3W	Nasopharynx	51 (42–59)	55	54	22	95	0	0	36	81	0	59	58
		B	116	Chemotherapy		53 (46.5–61)	46	62	11	105	0	0	39	77	0	58	58
PDR001 (NCT:NCT02605967)	Even 2021 (44)	A	82	Spartalizumab 400 mg Q4W	Nasopharynx	51 (21–74)	-	-	12	70	0	-	35	45	2	-	-
		B	40	Chemotherapy		50 (26–78)	-	-	2	37	1	-	11	28	0	-	-
EAGLE (NCT: 02369874)	Ferris 2020 (41)	A	240	Durvalumab 10 mg/kg Q2W	Oral cavity, opharynx, hypopharynx	59 (24–84)	68	172	198	35	-	5	62	178	-	194	46
		B	249	Cetuximab, taxane, methotrexate, or fluoropyrimidine		61 (22–82)	72	177	189	45	-	6	79	170	-	196	53

Subgroup A: Patients with platinum-refractory recurrent or metastatic head and neck squamous cell carcinoma who received PD1/PD-L1inhibitors; Subgroup B: Patients with platinum-refractory recurrent or metastatic head and neck squamous cell carcinoma who received chemotherapy or cetuximab; Q3W: Once every 3 weeks; Q4W: once every 4 weeks; Q2W: Once every 2 weeks; ECOG PS: eastern cooperative oncology group performance status.

### 3.3 Risk of bias

**FIGURE 2:**
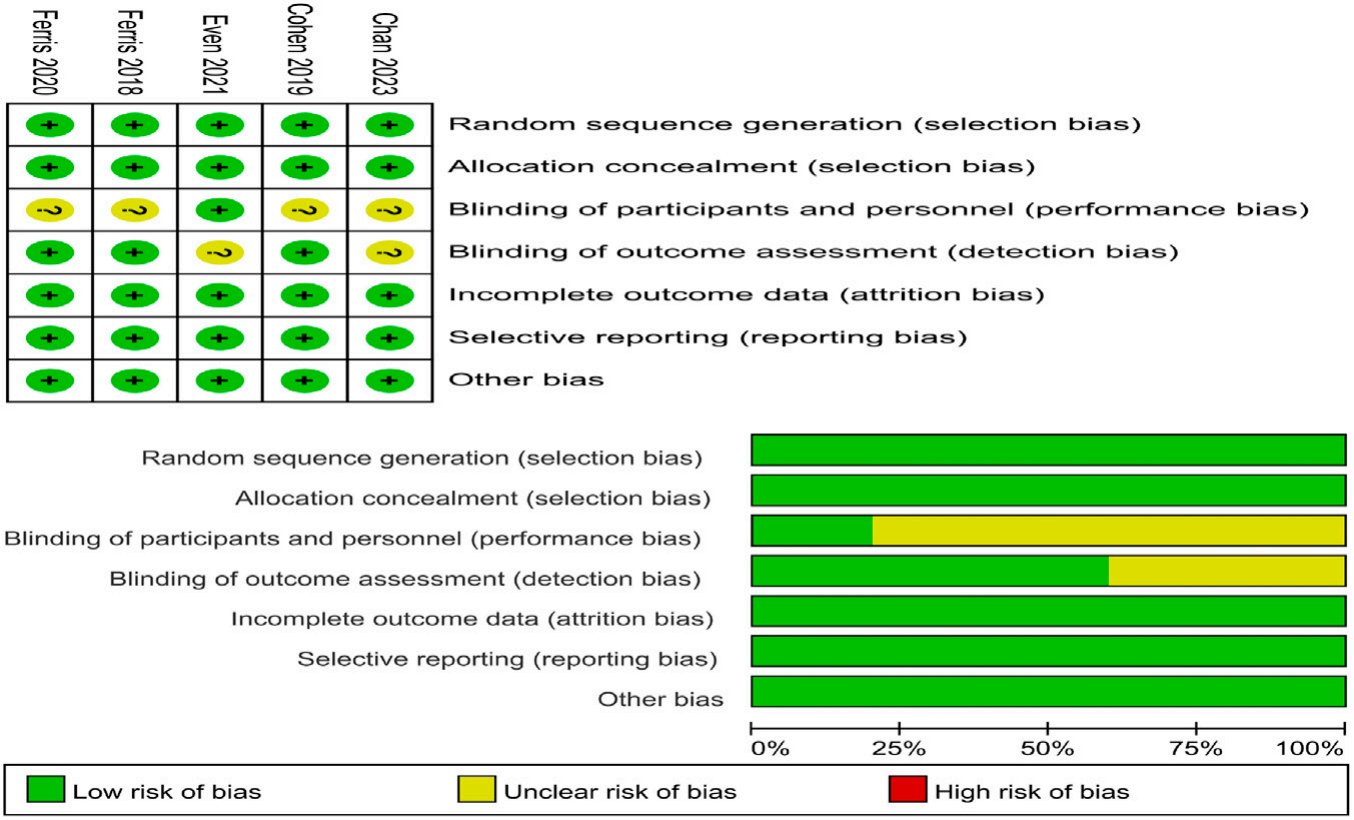
Risk of bias assessment summary.

Figure 2 provided a succinct summary of the results from the risk of bias assessment. Five RCTs produced a sufficient random sequence, Five studies reported appropriate allocation concealment, one studies clearly implemented participant blinding, three studies reported outcome assessor blinding, Five studies provided complete outcome data, Five studies did not engage in selective reporting, and Five studies did not exhibit any other bias (Figure 2).

### 3.4 Efficacy outcomes

#### 3.4.1 OS

**FIGURE 3:**
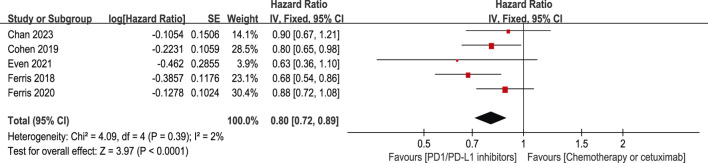
Forest plot of OS comparing PD-1/PD-L1 inhibitors versus chemotherapy or cetuximab.

All the 5 RCTs provided data regarding OS. (Ferris et al., 2018; Cohen et al., 2019a; Ferris et al., 2020; Even et al., 2021; Chan et al., 2023). PD-1/PD-L1 inhibitors was significantly superior to chemotherapy or cetuximab in terms of the median OS (HR: 0.80, 95%CI 0.72 to 0.89, P < 0.01, I^2^ = 2%) (Figure 3).

**FIGURE 4:**
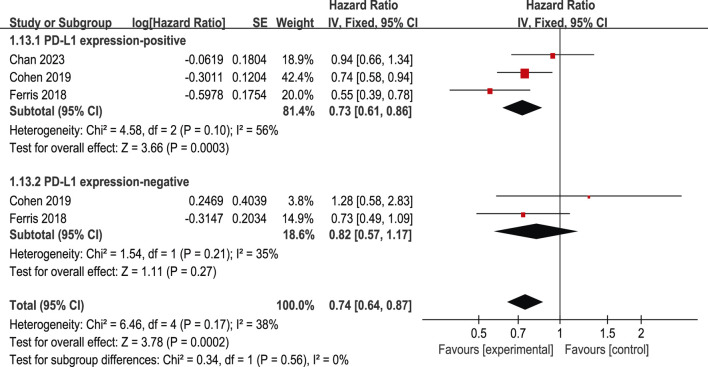
Forest plot of OS subgroup analysis by PD-L1 expression status.

Subgroup analysis regarding OS was conducted based on PD-L1 status. In the PD-L1 expression-positive subgroup, PD-1/PD-L1 inhibitors was significantly superior to chemotherapy or cetuximab in terms of OS (HR = 0.73, 95% CI: 0.61 to 0.86, P < 0.01, I^2^ = 38%). In the PD-L1 expression-negative subgroup, there was no statistically significant difference in terms of OS between the two groups (HR: 0.82, 95% CI: 0.57 to 1.17, P = 0.27) (Figure 4).

**FIGURE 5:**
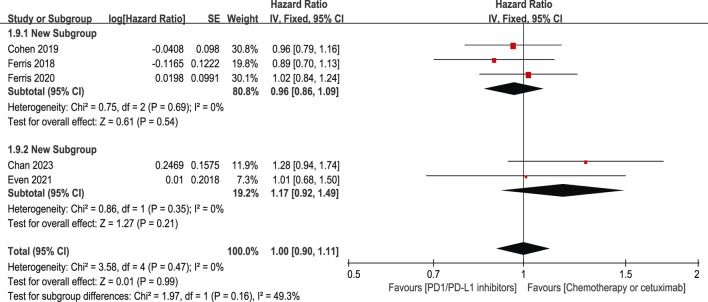
Forest plot of OS subgroup analysis by primary tumor site.

Subgroup analysis regarding OS was conducted based on the restriction status of primary tumor sites (Figure 5). Regarding NPC, PD-1/PD-L1 inhibitors was superior to chemotherapy or cetuximab, but there was no statistically significant difference in terms of OS (HR: 0.83, 95% CI: 0.64 to 1.08, P = 0.17, I^2^ = 18%). Regarding other HNSCC, PD-1/PD-L1 inhibitors was significantly superior to chemotherapy or cetuximab in terms of OS (HR = 0.79, 95% CI: 0.70 to 0.89, P < 0.01, I^2^ = 27%).

**FIGURE 6:**
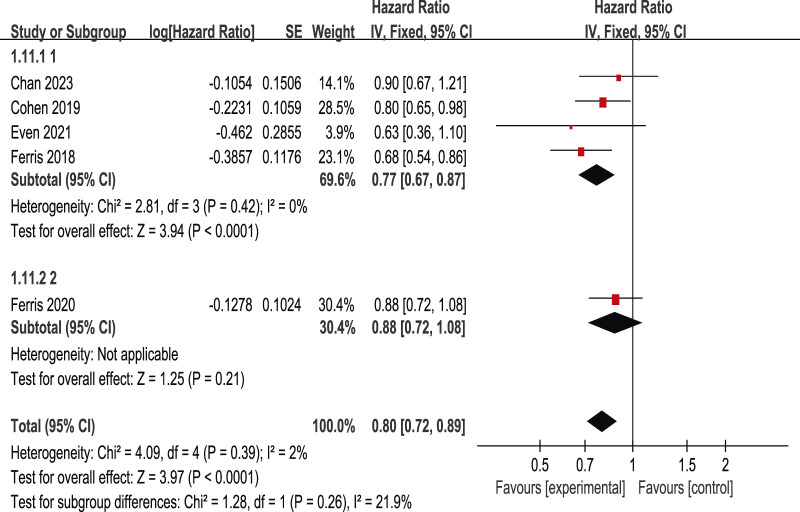
Forest plot of OS subgroup analysis by type of PD-1/PD-L1 inhibitor.

Subgroup analysis regarding OS was conducted based on type of PD-1/PD-L1 inhibitor (Figure 6). In the PD-L1 inhibitor subgroup, PD-L1 inhibitors was superior to chemotherapy or cetuximab in terms of OS (HR = 0.77, 95% CI: 0.67 to 0.87, P < 0.01, I^2^ = 27%). In the PD-1 inhibitor subgroup, there was no statistically significant difference in terms of OS between the two groups (HR: 0.88, 95% CI: 0.72 to 1.08, P = 0.21).

#### 3.4.2 PFS

**FIGURE 7:**
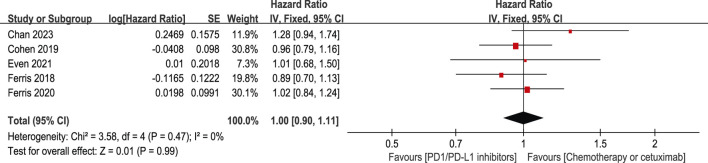
Forest plot of PFS comparing PD-1/PD-L1 inhibitors versus standard therapy.

All the 5 RCTs provided data regarding PFS (Ferris et al., 2018; Cohen et al., 2019a; Ferris et al., 2020; Even et al., 2021; Chan et al., 2023). There was no statistically significant difference between the PD-1/PD-L1 inhibitor group and the standard treatment group in terms of PFS (HR: 1.00, 95%CI: 0.90 to 1.11, P = 0.99, I^2^ = 0%) (Figure 7).

**FIGURE 8:**
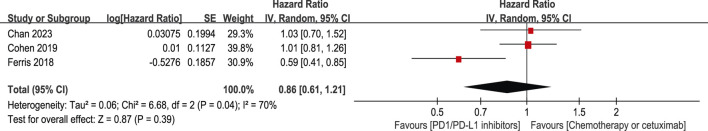
Forest plot of PFS subgroup analysis by PD-L1 expression status.

PFS subgroup analysis was conducted based on PD-L1 status. In the PD-L1 expression-positive subgroup, there was no statistically significant difference in terms of PFS between the two groups (HR: 0.86, 95%CI: 0.61 to 1.21, P = 0.39, I^2^ = 70%) (Figure 8). However, the included RCTs did not provide detailed data of the PD-L1 expression-negative subgroup. Sensitive analysis showed that the outcomes were stable (Supplementary Figure S1).

#### 3.4.3 ORR

**FIGURE 9:**
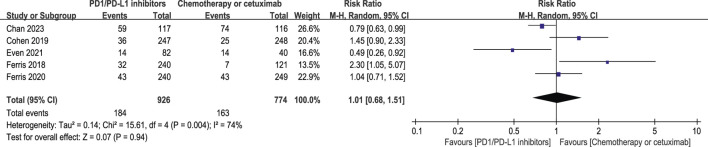
Forest plot of ORR comparing PD-1/PD-L1 inhibitors versus standard therapy.

All the 5 RCTs provided data regarding ORR (Ferris et al., 2018; Cohen et al., 2019a; Ferris et al., 2020; Even et al., 2021; Chan et al., 2023). There was no statistically significant difference in ORR between the PD-1/PD-L1 inhibitor group and the standard treatment group (RR = 1.01, 95% CI: 0.68 to 1.51, P = 0.94, I^2^ = 74%) (Figure 9). Sensitive analysis showed that the outcomes were stable (Supplementary Figure S2).

### 3.5 Safety outcomes

**FIGURE 10:**
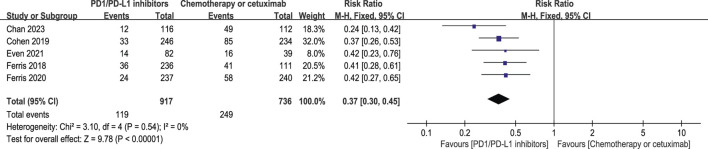
Forest plot of grade 3-5 trAEs comparing PD-1/PD-L1 inhibitors versus standard therapy.

All the 5 RCTs provided data regarding grade 3–5 trAEs (Ferris et al., 2018; Cohen et al., 2019a; Ferris et al., 2020; Even et al., 2021; Chan et al., 2023). With regards to grade 3–5 trAEs, patients in the PD-1/PD-L1 inhibitors group was significant lower than that of the chemotherapy or cetuximab group (RR = 0.37, 95% CI: 0.30 to 0.45, P < 0.01, I^2^ = 0%) (Figure 10).

**FIGURE 11:**
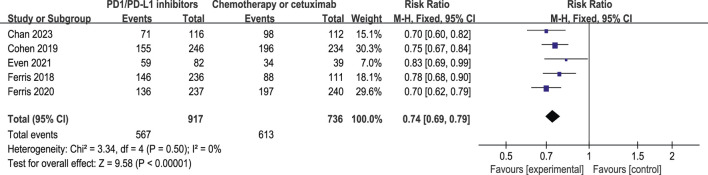
Forest plot of any-grade trAEs comparing PD-1/PD-L1 inhibitors versus standard therapy.

All the 5 RCTs provided data regarding any grade trAEs (Ferris et al., 2018; Cohen et al., 2019a; Ferris et al., 2020; Even et al., 2021; Chan et al., 2023). With regards to any grade trAEs, patients in the PD-1/PD-L1 inhibitors group was significant lower than that of the chemotherapy or cetuximab group (RR = 0.74, 95% CI: 0.69 to 0.79, P < 0.00001, I^2^ = 0%) (Figure 11).

### 3.6 Publication bias assessment

The evaluation of publication bias was conducted using funnel plots. The bilateral symmetric funnel plot regarding OS, PFS, ORR, grade 3–5 trAEs and any grade trAEs (Supplementary Figures S3–S7) did not reveal any significant evidence of publication bias.

### 3.7 Evidence certainty

**FIGURE 12:**
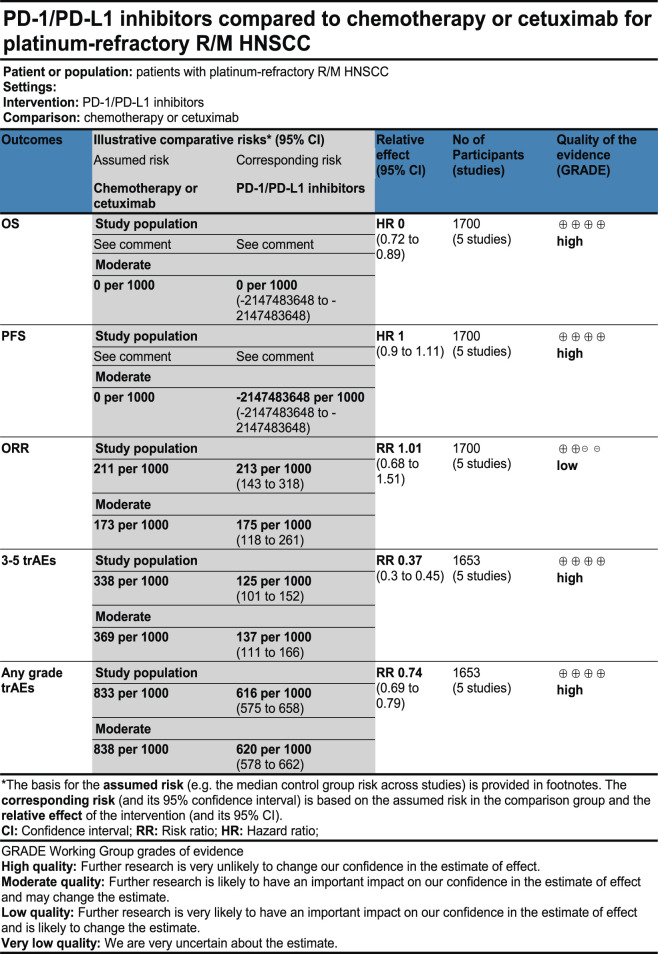
Certainty of evidence using GRADEpro GDT.

The certainty of evidence assessed for the various outcomes as per GRADE (Grading of Recommendations, Assessment, Development and Evaluations) criteria were of low certainty category (Figure 12).

## 4 Discussion

Compared with a prior meta-analysis of three RCTs (Zhu et al., 2021), the present meta-analysis of five RCTs (N = 1,700) offers key contributions: larger sample size, PD-L1 subgroup analysis showing OS benefit in PD-L1-positive patients, first subgroup analysis by tumor site (NPC vs. non-NPC), and updated safety data. These findings provide more robust evidence for PD-1/PD-L1 inhibitors in platinum-refractory R/M HNSCC.

Although NPC and non-NPC HNSCC differ in etiology and tumor microenvironment (Wong et al., 2021), both are squamous cell carcinomas arising from the head and neck region, and PD-1/PD-L1 inhibitors have been approved by the FDA for platinum-refractory recurrent/metastatic HNSCC regardless of primary tumor site (Larkins et al., 2017). Thus, from clinical and regulatory standpoints, pooling these populations is justified. To address potential biological heterogeneity, a pre-specified subgroup analysis by primary tumor site was performed (Figure 5), which showed consistent OS benefit direction across both subgroups. The NPC subgroup did not reach statistical significance, likely due to limited sample size (only two trials, n = 199).

This meta-analysis evaluated the efficacy and safety of PD-1/PD-L1 inhibitors versus chemotherapy or cetuximab in platinum-refractory R/ M HNSCC. This meta-analysis indicates that PD-1/PD-L1 inhibitors can enhance OS in platinum-refractory R/M HNSCC, particularly in populations with high PD-L1 expression. Furthermore, the incidence of grade 3–5 trAEs and any grade trAEs is lower, with no significant improvement observed in PFS or ORR. Consequently, PD-1/PD-L1 inhibitors are effective for platinum-refractory R/M HNSCC, particularly in populations with high PD-L1 expression, and are safer compared to standard treatments.

Subgroup analysis was conducted based on PD-L1 expression level. While PD-L1 expression level was positive, PD-1/PD-L1 inhibitors were superior to chemotherapy or cetuximab regarding OS, but there was no statistically significant difference between the two groups while PD-L1 expression level was negative. In addition, subgroup analysis regarding PFS revealed that PD-1/PD-L1 inhibitors provide longer PFS than chemotherapy or cetuximab, though with no statistically difference. Besides, the KEYNOTE-040 trial (Cohen et al., 2019a) reported that pembrolizumab provided superior PFS than chemotherapy or cetuximab while PD-L1 tumor scale score was higher than 50%. PD-1/PD-L1 inhibitors are likely to be a more efficacious treatment alternative, potentially attributable to the activation of the immune system and alterations in the tumor microenvironment (Pai et al., 2016; Ferris, 2015). PD-1 downregulates and suppresses T cells by binding to its ligand, PD-L1, enabling tumor cells to evade the attack of the immune system (Dong et al., 2002). Additionally, the ligands of PD-1 are also expressed in HNSCC (Parry et al., 2005). Research indicates that in HNSCC, upregulated PD-L1 attenuates T-cell cytotoxicity against tumor cells (Pai et al., 2016; Ferris et al., 2016; Seiwert et al., 2016). Simultaneously, the upregulated PD-L1 offers more targets for inhibitors (Pai et al., 2016). By hindering the PD-1/PD-L1 interaction, these inhibitors reactivate T cells to exert anti-tumor effects, resulting in better overall survival than conventional chemotherapy and cetuximab. Since NPC has distinct biological and clinical characteristics compared to other HNSCC, subgroup analysis regarding OS was conducted based on the restriction status of primary tumor sites (Figure 5). Regarding other HNSCC, PD-1/PD-L1 inhibitors was significantly superior to chemotherapy or cetuximab. However, regarding NPC, PD-1/PD-L1 inhibitors was superior to chemotherapy or cetuximab, but there was no statistically significant difference. These findings demonstrated potential benefit signals of PD-1/PD-L1 inhibitors in the treatment of NPC, which align with the immunogenic characteristics of this tumor. PD-1 and PD-L1 inhibitors target the same immune checkpoint pathway and are recommended as a class in clinical guidelines, supporting their pooled analysis (Network, 2018). The significant OS benefit for PD-L1 inhibitors versus the non-significant finding for PD-1 inhibitors is likely due to insufficient statistical power (1,260 vs. 347 patients), not a genuine lack of efficacy, and head-to-head comparisons are warranted. HPV and EBV statuses are important predictive biomarkers (Chung and Gillison, 2009), but quantitative subgroup analyses could not be performed due to incomplete data in three of five RCTs (Ferris et al., 2018; Cohen et al., 2019a; Even et al., 2021); future trials should incorporate viral status stratification.

In terms of ORR, the results show no statistical difference between the two groups. We failed to performed subgroup analysis based on PD-L1 expression level since no relative data was reported. Results from the CheckMate 141 Phase III trial indicated that HPV-positive patients receiving PD-1/PD-L1 inhibitors exhibited a better ORR, while the difference lacked statistical significance, and naveromab demonstrated an ORR more than double that of conventional treatment (13.3% vs 5.8%) (Yen et al., 2020). The HPV status might serve as a possible predictive biomarker for immune checkpoint inhibitor therapy in patients with R/M HNSCC (Wang et al., 2019). The significant OS benefit without corresponding PFS or ORR improvements is well-recognized in immunotherapy trials, reflecting the time-dependent T-cell reactivation process, which can cause delayed responses, pseudoprogression, late curve separation indicating durable disease control (Wolchok et al., 2009), and the inability of early endpoints to capture prolonged survival from stable disease (O’Meara et al., 2023). Therefore, OS remains the most reliable endpoint for assessing immunotherapeutic efficacy in platinum-refractory R/M HNSCC.

In terms of safety, the incidence of grade 3–5 trAEs and any grade trAEs in the PD-1/PD-L1 inhibitor group was lower than that in the standard treatment group, indicating that the PD-1/PD-L1 inhibitor was safer than the standard treatment. The trAEs in the PD-1/PD-L1 inhibitor group mainly include hypothyroidism, fatigue, diarrhea, rash, anemia, and nausea, *etc.* The adverse reactions in the standard treatment group mainly include fatigue, rash, anemia, stomatitis, neutropenia, and alopecia, *etc.* (Cohen et al., 2019a) The incidence of adverse reactions of PD-1/PD-L1 inhibitors is lower and more tolerable than that of standard treatment, which will help improve patients’ compliance and tolerance to treatment. For grade 3 and above adverse responses, symptomatic treatment should be prioritized prior to confirming disease progression, initiating alternative therapy, or encountering unacceptable toxicity, and whether the drug can be discontinued after a reasonable clinical evaluation (Ferris et al., 2020). Of note, the present analysis focused on overall trAEs as reported in the original RCTs, rather than specifically on immune-related adverse events (irAEs), because the definition and capture of irAEs were not standardized or consistently reported across the included trials, precluding a reliable pooled analysis. Nevertheless, irAEs, such as pneumonitis, colitis, and endocrinopathies (e.g., hypothyroidism), are clinically important and unique to checkpoint inhibitors, warranting distinct monitoring and management strategies in clinical practice (Postow et al., 2018).

A previous meta-analysis consists of 3 overlapping RCTs have compared PD-1/PD-L1 inhibitors versus chemotherapy or cetuximab for platinum-refractory R/M HNSCC (Chung and Gillison, 2009). This updated meta-analysis included five RCTs, and the larger sample size might strengthen the validity, reliability, and clinical applicability of the results. Besides, the previous meta-analysis failed to perform subgroup analyses regarding survival based on PD-L1 expression. Subgroup analyses based on PD-L1 expression indicated that PD-1/PD-L1 inhibitors were more effective particularly in populations with high PD-L1 expression regarding OS. Therefore, the findings offered updated evidence-based support for the utilization of PD-1/PD-L1 inhibitors in platinum-refractory R/M HNSCC. Besides, all the studies included were of a randomized controlled trial nature and resulted in high GRADE evidence grading.

However, This study had certain limitations. First, despite comprehensive searches across four databases, our literature search approach might have exhibited deficiencies, potentially resulting in the exclusion of relevant material and inaccessible data from completed but unpublished research, which could inflate effect sizes. Several literary sources were deficient in essential data, and despite efforts to reach out to authors, a significant amount of data remained inaccessible. Second, only five RCTs were included due to our rigorous inclusion criteria. The limited number of included studies, coupled with potential data gaps or methodological constraints, poses challenges to the robustness and broader applicability of our findings. Third, PD-L1 expression was assessed using different scoring systems (CPS vs. TPS) and antibody clones across trials, though all used clinically validated assays with a cutoff of CPS ≥1 or TPS ≥1% (Ferris et al., 2018). This aligns with clinical practice but may introduce heterogeneity (Cohen et al., 2019a), and the potential impact on subgroup outcomes remains a limitation. Fourth, with fewer than 10 included studies, the power of funnel plot-based tests is limited; therefore, these results should be interpreted cautiously and do not definitively exclude publication bias. Fifth, control arms varied substantially across the five RCTs, but due to the limited number of studies, subgroup analyses based on specific regimens could not be performed; this heterogeneity remains a major limitation that may have influenced the pooled estimates (Cumpston et al., 2023). Sixth, we performed leave-one-out sensitivity analyses for outcomes with I^2^ >50%, the limited number of included studies precludes more robust meta-regression analyses.

Consequently, the results should be interpreted with caution. Furthermore, though subgroup analysis regarding OS was conducted based on PD-L1 status, primary tumor sites or type of PD-1/PD-L1 inhibitor), we were unable to manage confounding variables, including of varying inclusion criteria, population disparities, HPV status and the regimens in the controlled group, which may lead to study heterogeneity and bias. Well planned RCTs are necessary for future prospective validation of these findings. Real-world studies are also recommended to verify the observed advantages of PD-1/PD-L1 inhibitors.

Future research directions beyond PD-L1 expression should include tumor mutational burden (TMB), immune gene signatures, tumor microenvironment profiling (e.g., CD8^+^ T cell infiltration), and circulating biomarkers (e.g., ctDNA, exosomes) (Gutiérrez Calderón et al., 2021). The role of viral status (HPV/EBV) as a predictive biomarker warrants prospective stratification in trial design. Additionally, combining PD-1/PD-L1 inhibitors with novel agents targeting other immune checkpoints (e.g., TIGIT, LAG-3) or the tumor vasculature may further improve outcomes in platinum-refractory R/M HNSCC (Chen et al., 2023).

In conclusion, the findings demonstrated that PD-1/PD-L1 inhibitors were effective for platinum-refractory R/M HNSCC particularly in populations with high PD-L1 expression, and are safer than chemotherapy or cetuximab.

## Data Availability

The datasets presented in this study can be found in online repositories. The names of the repository/repositories and accession number(s) can be found in the article/Supplementary Material.
