# Supplementary material for: Efficacy and safety of PD-1/PD-L1 inhibitors versus chemotherapy or cetuximab in the treatment of platinum-refractory recurrent or metastatic head and neck squamous cell carcinoma: a meta-analysis
Source: Front Pharmacol. 2026 Jul 31;17:1675407. doi: 10.3389/fphar.2026.1675407 (PMC13473282; doi:10.3389/fphar.2026.1675407)
Supplement: Supplementary file 1 [file DataSheet1.pdf]

PubMed		
No.	Query	Results
1	((PD-1 inhibitor[Title/Abstract]) OR (Pembrolizumab[Title/Abstract])) OR (SCH-900475[Title/Abstract])) OR (Lambrolizumab[Title/Abstract])) OR (MK-3475[Title/Abstract])) OR (Keytruda[Title/Abstract])) OR (Nivolumab[Title/Abstract])) OR (Opdivo[Title/Abstract])) OR (ONO-4538[Title/Abstract])) OR (ONO 4538[Title/Abstract])) OR (ONO4538[Title/Abstract])) OR (MDX-1106[Title/Abstract])) OR (MDX 1106[Title/Abstract])) OR (MDX1106[Title/Abstract])) OR (BMS-936558[Title/Abstract])) OR (BMS 936558[Title/Abstract])) OR (BMS936558[Title/Abstract])) OR (Toripalimab[Title/Abstract])) OR (Tislelizumab[Title/Abstract])) OR (BGB-A317[Title/Abstract])) OR (Camrelizumab[Title/Abstract])) OR (carrelizumab[Title/Abstract])) OR (SHR-1210[Title/Abstract])) OR (SHR 1210[Title/Abstract])) OR (GLS-010[Title/Abstract])) OR (Cemiplimab[Title/Abstract])) OR (REGN2810[Title/Abstract])) OR (Sintilimab[Title/Abstract])) OR (IBI308[Title/Abstract])) OR (Zimberelimab[Title/Abstract])) OR (Prolgolimab[Title/Abstract])) OR (Dostarlimab[Title/Abstract])) OR (Jemperli[Title/Abstract])) OR (dostarlimab-gxly[Title/Abstract])) OR (TSR-042[Title/Abstract])) OR (PD-L1 inhibitor[Title/Abstract])) OR (Atezolizumab[Title/Abstract])) OR (anti-PDL1[Title/Abstract])) OR (immunoglobulin G1, anti-(human CD antigen CD274) (human monoclonal heavy chain), disulfide with human monoclonal kappa-chain, dimer[Title/Abstract])) OR (MPDL3280A[Title/Abstract])) OR (MPDL-3280A[Title/Abstract])) OR (Tecentriq[Title/Abstract])) OR (RG7446[Title/Abstract])) OR (RG-7446[Title/Abstract])) OR (Durvalumab[Title/Abstract])) OR (MEDI4736[Title/Abstract])) OR (MEDI-4736[Title/Abstract])) OR (Imfinzi[Title/Abstract])) OR (Avelumab[Title/Abstract])) OR (MSB0010682[Title/Abstract])) OR (bavencio[Title/Abstract])) OR (MSB0010718C[Title/Abstract])) OR (MSB-0010718C[Title/Abstract])) OR (PD-1 Inhibitors[Title/Abstract])) OR (PD 1 Inhibitors[Title/Abstract])) OR (PD-1 Inhibitor[Title/Abstract])) OR (Inhibitor, PD-1[Title/Abstract])) OR (PD 1 Inhibitor[Title/Abstract])) OR (Programmed Cell Death Protein 1 Inhibitor[Title/Abstract])) OR (Programmed Cell Death Protein 1 Inhibitors[Title/Abstract])) OR (PD-L1 Inhibitors[Title/Abstract])) OR (PD L1 Inhibitors[Title/Abstract])) OR (PD-L1 Inhibitor[Title/Abstract])) OR (PD L1 Inhibitor[Title/Abstract])) OR (Programmed Death-Ligand 1 Inhibitors[Title/Abstract])) OR (Programmed Death Ligand 1 Inhibitors[Title/Abstract])) OR (PD-1-PD-L1 Blockade[Title/Abstract])) OR (Blockade, PD-1-PD-L1[Title/Abstract])) OR (PD 1 PD L1 Blockade[Title/Abstract])	34,516 results

2	("head and neck"[Title/Abstract] OR "oropharynx"[Title/Abstract] OR "oropharyngeal"[Title/Abstract] OR "nose"[Title/Abstract] OR "nasopharynx"[Title/Abstract] OR "nasopharyngeal"[Title/Abstract] OR "nasal sinus"[Title/Abstract] OR "nasal cavity"[Title/Abstract] OR "oral cavity"[Title/Abstract] OR "hypopharynx"[Title/Abstract] OR "hypopharyngeal"[Title/Abstract] OR "oral"[Title/Abstract] OR "laryngopharynx"[Title/Abstract] OR "larynx"[Title/Abstract] OR "laryngopharyngeal"[Title/Abstract] OR "laryngeal"[Title/Abstract] OR "pharyngeal"[Title/Abstract] OR "tongue"[Title/Abstract] OR "tonsil"[Title/Abstract] OR "tonsillar"[Title/Abstract] OR "cheek"[Title/Abstract] OR "palatal"[Title/Abstract] OR "paranasal sinuses"[Title/Abstract] OR "buccal"[Title/Abstract] OR "lip"[Title/Abstract]) AND ("cancer"[Title/Abstract] OR "tumor"[Title/Abstract] OR "neoplasm"[Title/Abstract] OR "carcinoma"[Title/Abstract])	263，874 results
3	(((((platinum resistant[Title/Abstract]) OR (platinum-resistant[Title/Abstract]) OR (platinum-refractory[Title/Abstract]) OR (platinum refractory[Title/Abstract]) OR (platinum-insensitive[Title/Abstract]) OR (platinum insensitive[Title/Abstract])	2,800 results
4	(((((Metastatic[Title/Abstract]) OR (metastatic[Title/Abstract])) OR (METASTATIC[Title/Abstract])) OR (Advanced[Title/Abstract])) OR (advanced[Title/Abstract])) OR (ADVANCED[Title/Abstract])	971，847 results
5	1 AND 2 AND 3 AND 4	78 results

Web of science		
No.	Query	Results
1	(((TS=(PD-1 inhibitor)) OR TS=(PD-1 Inhibitors)) OR TS=(PD 1 Inhibitors)) OR TS=(PD-1 Inhibitor)) OR TS=(Inhibitor, PD-1)) OR TS=(PD 1 Inhibitor)) OR TS=(Programmed Cell Death Protein 1 Inhibitor)) OR	141，862 results

	TS=(Programmed Cell Death Protein 1 Inhibitors)) OR TS=(Pembrolizumab)) OR TS=(SCH-900475)) OR TS=(Lambrolizumab)) OR TS=(MK-3475)) OR TS=(Keytruda)) OR TS=(Nivolumab)) OR TS=(Opdivo)) OR TS=(ONO-4538)) OR TS=(ONO 4538)) OR TS=(ONO4538)) OR TS=(MDX-1106)) OR TS=(MDX 1106)) OR TS=(MDX1106)) OR TS=(BMS-936558)) OR TS=(BMS 936558)) OR TS=(BMS936558)) OR TS=(Toripalimab)) OR TS=(Tislelizumab)) OR TS=(BGB-A317)) OR TS=(Camrelizumab)) OR TS=(carrelizumab)) OR TS=(SHR-1210)) OR TS=(SHR 1210)) OR TS=(GLS-010)) OR TS=(Cemiplimab)) OR TS=(REGN2810)) OR TS=(Sintilimab)) OR TS=(IBI308)) OR TS=(IBI-308)) OR TS=(Zimberelimab)) OR TS=(Prolgolimab)) OR TS=(Dostarlimab)) OR TS=(Jemperli)) OR TS=(dostarlimab-gxly)) OR TS=(TSR-042)) OR TS=(GSK4057190)) OR TS=(PD-L1 inhibitor)) OR TS=(PD-L1 Inhibitors)) OR TS=(PD L1 Inhibitors)) OR TS=(PD-L1 Inhibitor)) OR TS=(PD L1 Inhibitor)) OR TS=(Programmed Death-Ligand 1 Inhibitors)) OR TS=(Programmed Death Ligand 1 Inhibitors)) OR TS=(PD-1-PD-L1 Blockade)) OR TS=(Blockade, PD-1-PD-L1)) OR TS=(PD 1 PD L1 Blockade)) OR TS=(Atezolizumab)) OR TS=(anti-PDL1)) OR TS=(immunoglobulin G1, anti-(human CD antigen CD274) (human monoclonal MDPL3280a heavy chain), disulfide with human monoclonal MDPL3280a kappa-chain, dimer)) OR TS=(MPDL3280A)) OR TS=(MPDL-3280A)) OR TS=(Tecentriq)) OR TS=(RG7446)) OR TS=(RG-7446)) OR TS=(Durvalumab)) OR TS=(MEDI4736)) OR TS=(MEDI-4736)) OR TS=(Imfinzi)) OR TS=(Avelumab)) OR TS=(MSB-0010682)) OR TS=(MSB0010682)) OR TS=(bavencio)) OR TS=(MSB0010718C)) OR TS=(MSB-0010718C)	
2	(TS=(head and neck OR oropharynx OR oropharyngeal OR nose OR nasopharynx OR nasopharyngeal OR nasal sinus OR nasal cavity OR oral cavity OR hypopharynx OR hypopharyngeal OR oral OR laryngopharynx OR larynx OR laryngopharyngeal OR laryngeal OR pharyngeal OR tongue OR tonsil OR tonsillar OR cheek OR palatal OR paranasal sinuses OR buccal OR lip)) AND TS=(cancer OR tumor OR neoplasm OR carcinoma)	763,891 results
3	TS=(platinum resistant OR platinum-resistant OR platinum-refractory OR platinum refractory OR platinum-insensitive OR platinum insensitive)	27,111 results
4	TS=(Metastatic OR metastatic OR METASTATIC OR Advanced OR advanced OR ADVANCED)	4,122,331 results
5	1 AND 2 AND 3 AND 4	181 results

Embase

No.	Query	Results
1	'pd-1 inhibitors':ab,ti OR 'pd 1 inhibitors':ab,ti OR 'pd-1 inhibitor':ab,ti OR 'inhibitor, pd-1':ab,ti OR 'pd 1 inhibitor':ab,ti OR 'programmed cell death protein 1 inhibitors':ab,ti OR 'programmed cell death protein 1 inhibitor':ab,ti OR pembrolizumab:ab,ti OR 'sch 900475':ab,ti OR lambrolizumab:ab,ti OR 'mk 3475':ab,ti OR keytruda:ab,ti OR nivolumab:ab,ti OR opdivo:ab,ti OR 'ono 4538':ab,ti OR 'mdx 1106':ab,ti OR mdx1106:ab,ti OR 'bms 936558':ab,ti OR bms936558:ab,ti OR toripalimab:ab,ti OR 'bgb a317':ab,ti OR camrelizumab:ab,ti OR carrelizumab:ab,ti OR 'shr 1210':ab,ti OR 'gls 010':ab,ti OR cemiplimab:ab,ti OR regn2810:ab,ti OR sintilimab:ab,ti OR ibi308:ab,ti OR 'ibi 308':ab,ti OR zimberelimab:ab,ti OR prolgolimab:ab,ti OR dostarlimab:ab,ti OR jemperli:ab,ti OR 'dostarlimab gxly':ab,ti OR 'tsr 042':ab,ti OR gsk4057190:ab,ti OR 'pd-l1 inhibitors':ab,ti OR 'pd l1 inhibitors':ab,ti OR 'pd-l1 inhibitor':ab,ti OR 'pd l1 inhibitor':ab,ti OR 'programmed death-ligand 1 inhibitors':ab,ti OR 'programmed death ligand 1 inhibitors':ab,ti OR 'pd-1-pd-l1 blockade':ab,ti OR 'blockade, pd-1-pd-l1':ab,ti OR 'pd 1 pd l1 blockade':ab,ti OR atezolizumab:ab,ti OR 'anti pdl1':ab,ti OR ('immunoglobulin g1, anti-':ab,ti AND 'human cd antigen cd274':ab,ti AND 'human monoclonal mdpl3280a heavy chain':ab,ti AND ', disulfide with human monoclonal mdpl3280a kappa-chain, dimer':ab,ti) OR mpdl3280a:ab,ti OR 'mpdl 3280a':ab,ti OR tecentriq:ab,ti OR rg7446:ab,ti OR 'rg 7446':ab,ti OR durvalumab:ab,ti OR medi4736:ab,ti OR 'medi 4736':ab,ti OR imfinzi:ab,ti OR avelumab:ab,ti OR 'msb 0010682':ab,ti OR msb0010682:ab,ti OR bavencio:ab,ti OR msb0010718c:ab,ti OR 'msb 0010718c':ab,ti	79,706 results
2	('head and neck':ab,ti,kw OR 'oropharynx':ab,ti,kw OR 'oropharyngeal':ab,ti,kw OR 'nose':ab,ti,kw OR 'nasopharynx':ab,ti,kw OR 'nasopharyngeal':ab,ti,kw OR 'nasal sinus':ab,ti,kw OR 'nasal cavity':ab,ti,kw OR 'oral cavity':ab,ti,kw OR 'hypopharynx':ab,ti,kw OR 'hypopharyngeal':ab,ti,kw OR 'oral':ab,ti,kw OR 'laryngopharynx':ab,ti,kw OR 'larynx':ab,ti,kw OR 'laryngopharyngeal':ab,ti,kw OR 'laryngeal':ab,ti,kw OR 'pharyngeal':ab,ti,kw OR 'tongue':ab,ti,kw OR 'tonsil':ab,ti,kw OR 'tonsillar':ab,ti,kw OR 'cheek':ab,ti,kw OR 'palatal':ab,ti,kw OR 'paranasal sinuses':ab,ti,kw OR 'buccal':ab,ti,kw OR 'lip':ab,ti,kw) AND ('cancer':ab,ti,kw OR 'tumor':ab,ti,kw OR 'neoplasm':ab,ti,kw OR 'carcinoma':ab,ti,kw)	395，380 results
3	'platinum resistant':ab,ti OR 'platinum-resistant':ab,ti OR 'platinum-refractory':ab,ti OR 'platinum refractory':ab,ti OR 'platinum-insensitive':ab,ti OR 'platinum insensitive':ab,ti	6，025 results

4	metastatic:ab,ti OR advanced:ab,ti	1，501，547 results
5	1 AND 2 AND 3 AND 4	199 results

cochrane library		
No.	Query	Results
1	(PD-1Inhibitors OR PD-1 Inhibitors OR PD 1 Inhibitors OR PD-1 Inhibitor OR Inhibitor, PD-1 OR PD 1 Inhibitor OR Programmed Cell Death Protein 1 Inhibitor OR Programmed Cell Death Protein 1 Inhibitors OR Nivolumab OR Opdivo OR ONO-4538 OR ONO 4538 OR ONO4538 OR MDX-1106 OR MDX 1106 OR MDX1106 OR BMS-936558 OR BMS 936558 OR BMS936558 OR Pembrolizumab OR SCH-900475 OR lambrolizumab OR MK-3475 OR Keytruda OR Tislelizumab OR BGB-A317 OR Toripalimab OR Camrelizumab OR carrelizumab OR SHR-1210 OR SHR 1210 OR Sintilimab OR IBI 308 OR IBI308 OR IBI-308 OR Zimberelimab OR GLS-010 OR Prolgolimab OR Dostarlimab OR Jemperli OR dostarlimab-gxly OR TSR-042 OR GSK4057190 OR PD-L1 Inhibitors OR PD-L1 Inhibitors OR PD L1 Inhibitors OR PD-L1 Inhibitor OR PD L1 Inhibitor OR Programmed Death-Ligand 1 Inhibitors OR Programmed Death Ligand 1 Inhibitors OR PD-1 PD-L1 Blockade OR Blockade, PD-1 PD-L1 OR PD 1 PD L1 Blockade OR Atezolizumab OR anti-PDL1 OR immunoglobulin G1, anti-(human CD antigen CD274) (human monoclonal MDPL3280a heavy chain), disulfide with human monoclonal MDPL3280a kappa-chain, dimer OR MPDL3280A OR MPDL-3280A OR Tecentriq OR RG7446 OR RG-7446 OR Durvalumab OR MEDI4736 OR MEDI-4736 OR Imfinzi OR Avelumab OR MSB-0010682 OR MSB0010718C OR MSB-0010718C OR MSB0010682 OR bavencio):ti,ab,kw	18,073 results
2	('head and neck':ab,ti,kw OR 'oropharynx':ab,ti,kw OR 'oropharyngeal':ab,ti,kw OR 'nose':ab,ti,kw OR 'nasopharynx':ab,ti,kw OR 'nasopharyngeal':ab,ti,kw OR 'nasal sinus':ab,ti,kw OR 'nasal cavity':ab,ti,kw OR 'oral cavity':ab,ti,kw OR 'hypopharynx':ab,ti,kw OR 'hypopharyngeal':ab,ti,kw OR 'oral':ab,ti,kw OR 'laryngopharynx':ab,ti,kw OR 'larynx':ab,ti,kw OR 'laryngopharyngeal':ab,ti,kw OR 'laryngeal':ab,ti,kw OR 'pharyngeal':ab,ti,kw OR 'tongue':ab,ti,kw OR 'tonsil':ab,ti,kw OR 'tonsillar':ab,ti,kw OR 'cheek':ab,ti,kw OR 'palatal':ab,ti,kw OR 'paranasal sinuses':ab,ti,kw OR 'buccal':ab,ti,kw OR 'lip':ab,ti,kw) AND ('cancer':ab,ti,kw OR 'tumor':ab,ti,kw OR 'neoplasm':ab,ti,kw OR 'carcinoma':ab,ti,kw)	40，001 results

3	'platinum resistant':ab,ti,kw OR 'platinum-resistant':ab,ti,kw OR 'platinum-refractory':ab,ti,kw OR 'platinum refractory':ab,ti,kw OR 'platinum-insensitive':ab,ti,kw OR 'platinum insensitive':ab,ti,kw	1,672 results
4	(Advanced)ti,ab,kw OR (metastatic)ti,ab,kw OR (Metastatic)ti,ab,kw OR (metastatic)ti,ab,kw OR (METASTATIC)ti,ab,kw OR (Advanced)ti,ab,kw OR (advanced)ti,ab,kw OR (ADVANCED):ti,ab,kw	78,794 results
5	#1 AND #2 AND #3 AND #4	36 results
