# Supplementary material for: Efficacy and safety of PD-1/PD-L1 inhibitors versus chemotherapy or cetuximab in the treatment of platinum-refractory recurrent or metastatic head and neck squamous cell carcinoma: a meta-analysis
Source: Front Pharmacol. 2026 Jul 31;17:1675407. doi: 10.3389/fphar.2026.1675407 (PMC13473282; doi:10.3389/fphar.2026.1675407)
Supplement: Supplementary file 2 [file DataSheet2.pdf]

**Supplement Figure Legends**

**Supplement Figure 1. Sensitivity analysis for PFS in the PD-L1-positive subgroup.**
Leave-one-out sensitivity analysis was performed to assess the influence of each individual study on the pooled PFS outcome in the PD-L1-positive subgroup. The horizontal lines represent the pooled HRs and their 95% CIs after omitting each study sequentially. The analysis confirmed that no single study disproportionately influenced the overall pooled estimate, supporting the robustness of the primary PFS findings.

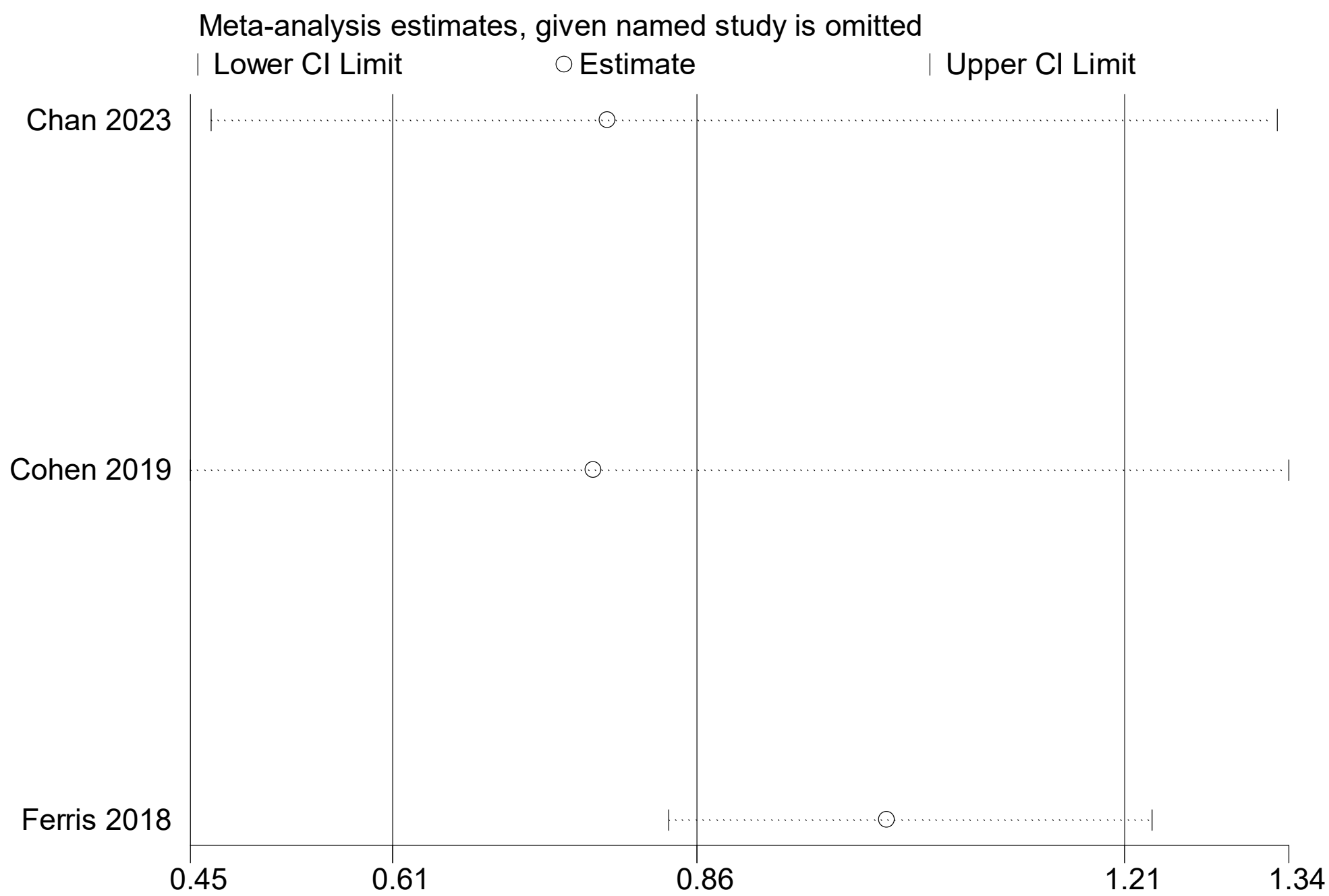


**Supplement Figure 2. Sensitivity analysis for ORR.**
Leave-one-out sensitivity analysis was conducted to evaluate the stability of the pooled ORR result. The diagram shows the pooled RRs and their 95% CIs after sequentially excluding each study. The analysis revealed that the exclusion of any single study did not materially alter the overall non-significant result (pooled RR approximately 1.0), confirming the stability of the meta-analysis.

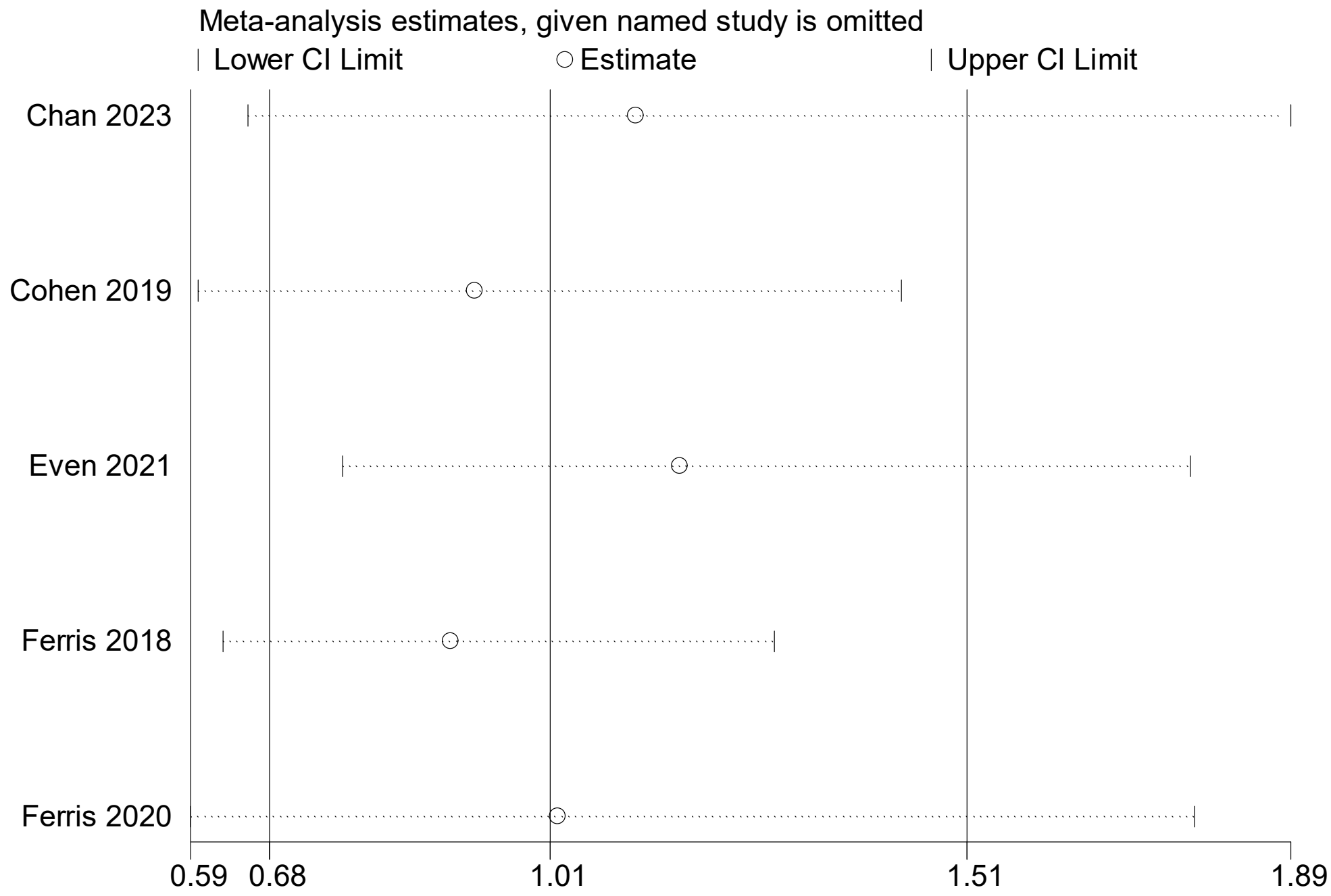


**Supplement Figure 3. Funnel plot for publication bias assessment of OS.**

A funnel plot was generated to assess potential publication bias among the five studies reporting OS. The plot shows the log of the hazard ratio (log[HR]) on the x-axis against its standard error (SE) on the y-axis. The symmetrical distribution of the points within the pseudo-95% confidence limits suggests no significant publication bias for the OS outcome.

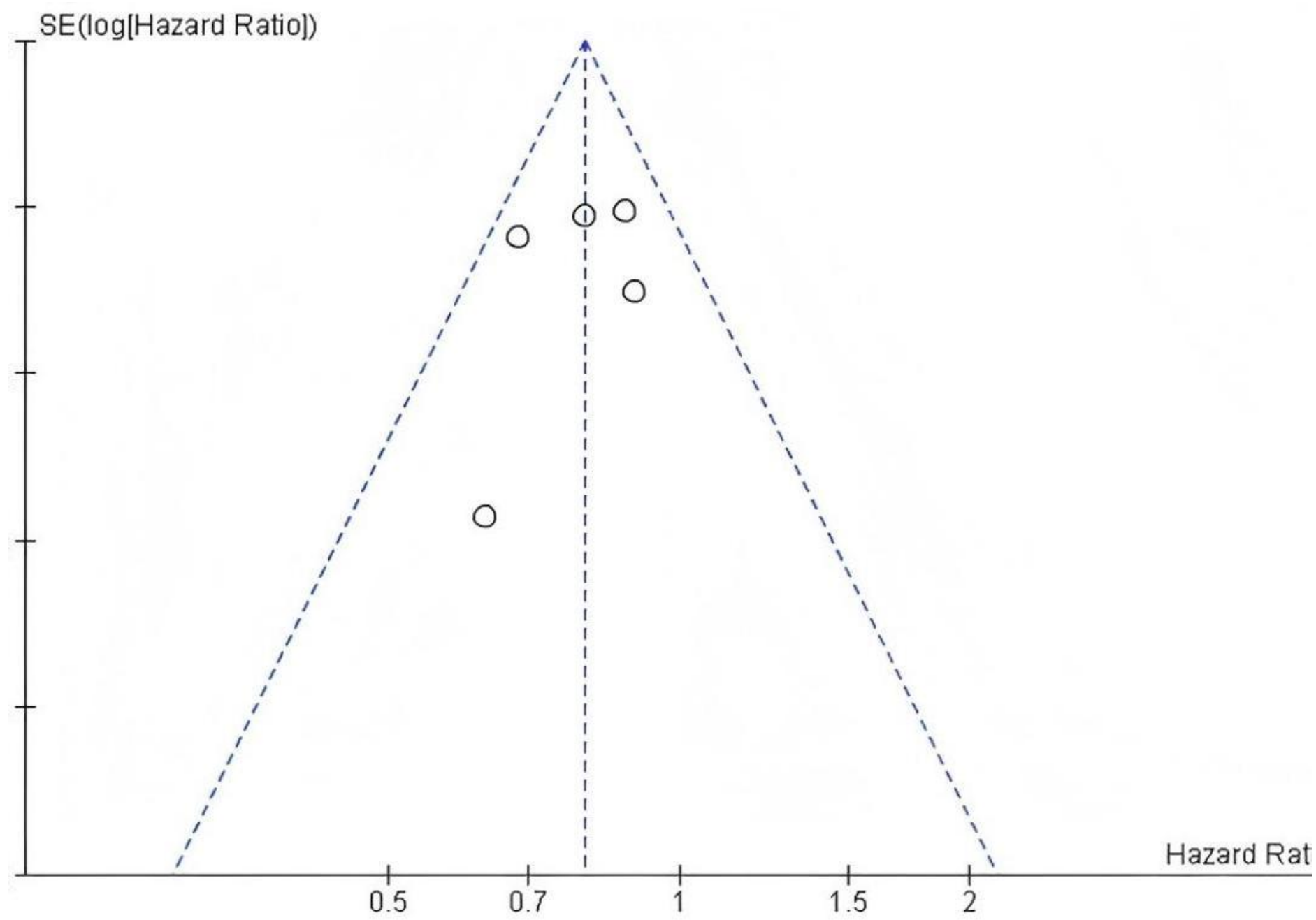

**Supplement Figure 4. Funnel plot for publication bias assessment of PFS.**
This funnel plot displays the distribution of included studies for the PFS outcome. The symmetrical scatter of the plotted points around the pooled effect estimate provides no strong visual evidence of publication bias for PFS.

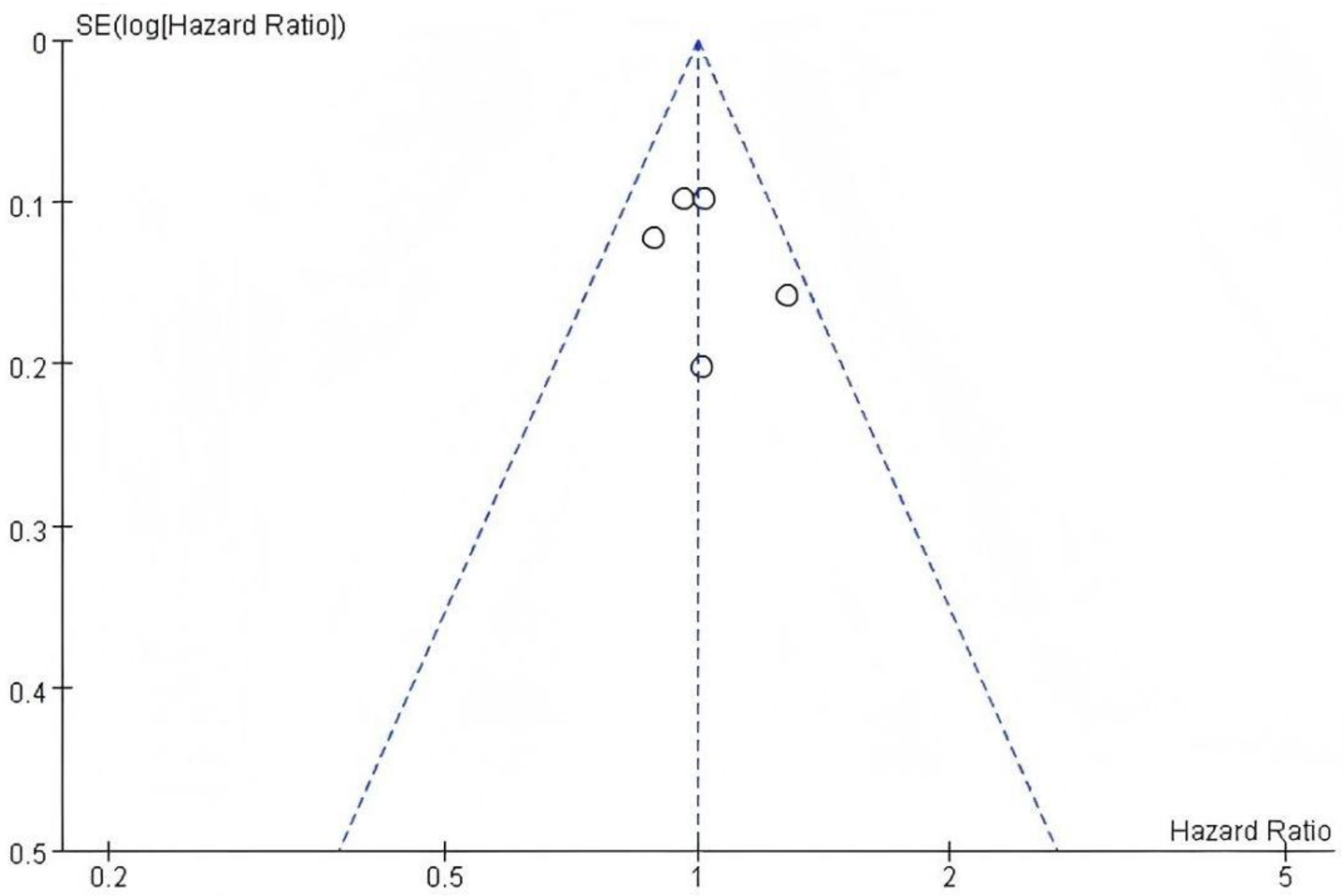


**Supplement Figure 5. Funnel plot for publication bias assessment of ORR.**
The funnel plot for ORR shows the log of the relative risk (log[RR]) plotted against its standard error. The symmetrical distribution of the studies suggests a low risk of publication bias for the ORR outcome.

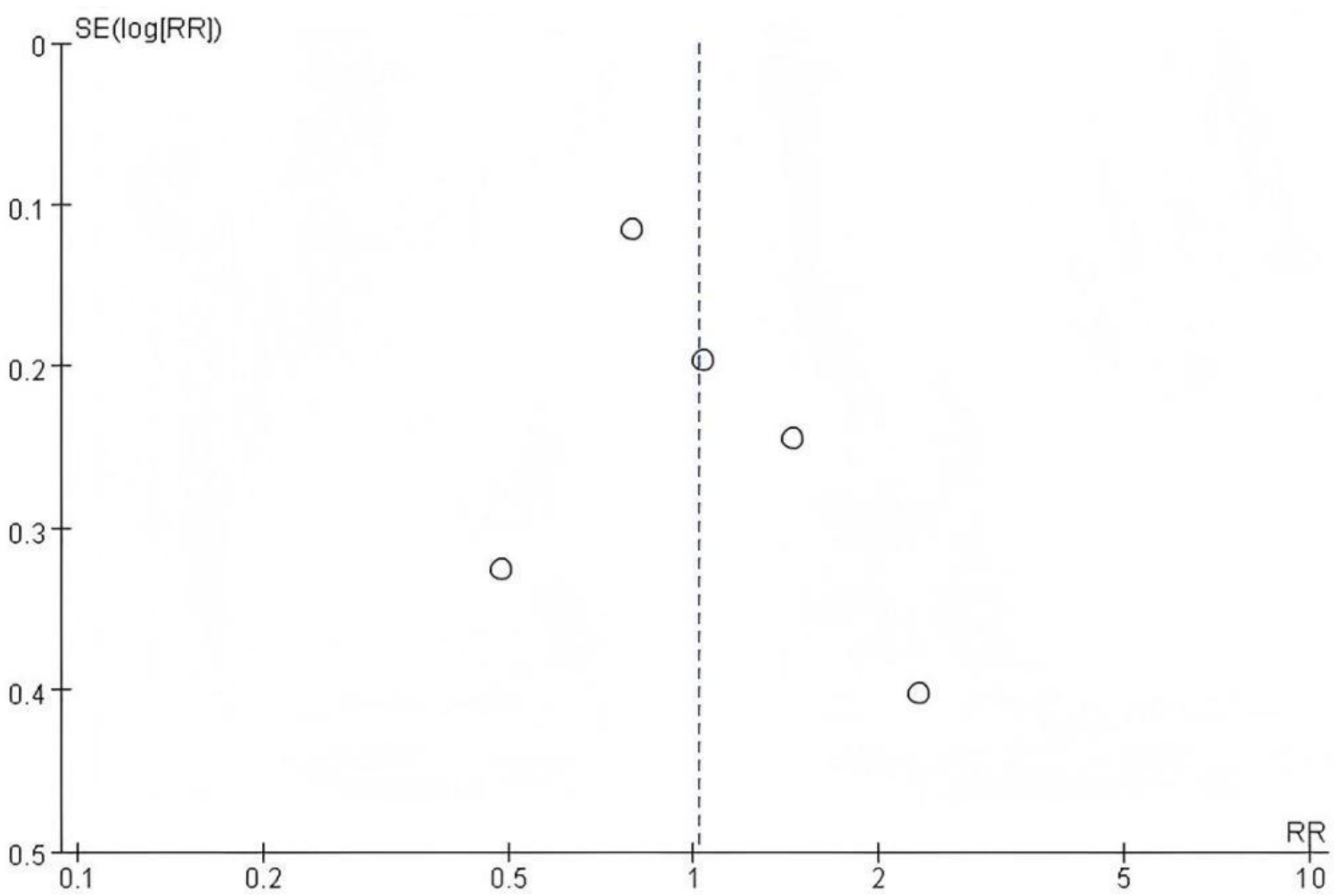

**Supplement Figure 6. Funnel plot for publication bias assessment of grade 3-5 trAEs.**

This funnel plot assessed publication bias for the outcome of grade 3-5 treatment-related adverse events. The symmetrical distribution of the points around the null effect line indicates no significant publication bias.

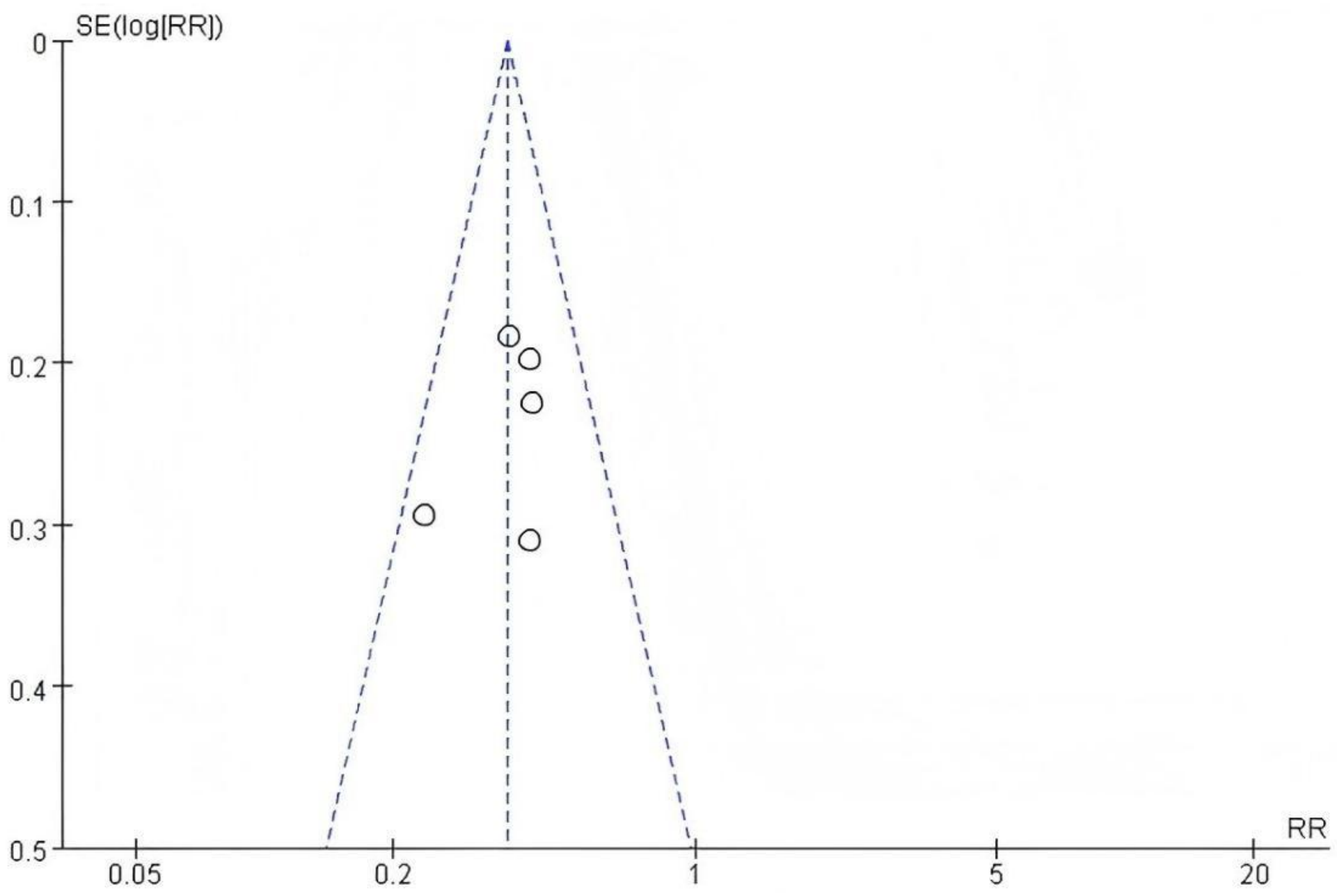


**Supplement Figure 7. Funnel plot for publication bias assessment of any-grade trAEs.**

This funnel plot evaluated publication bias for any-grade treatment-related adverse events. The visual symmetry of the plot suggests that publication bias is unlikely to have affected the pooled estimate for this outcome.

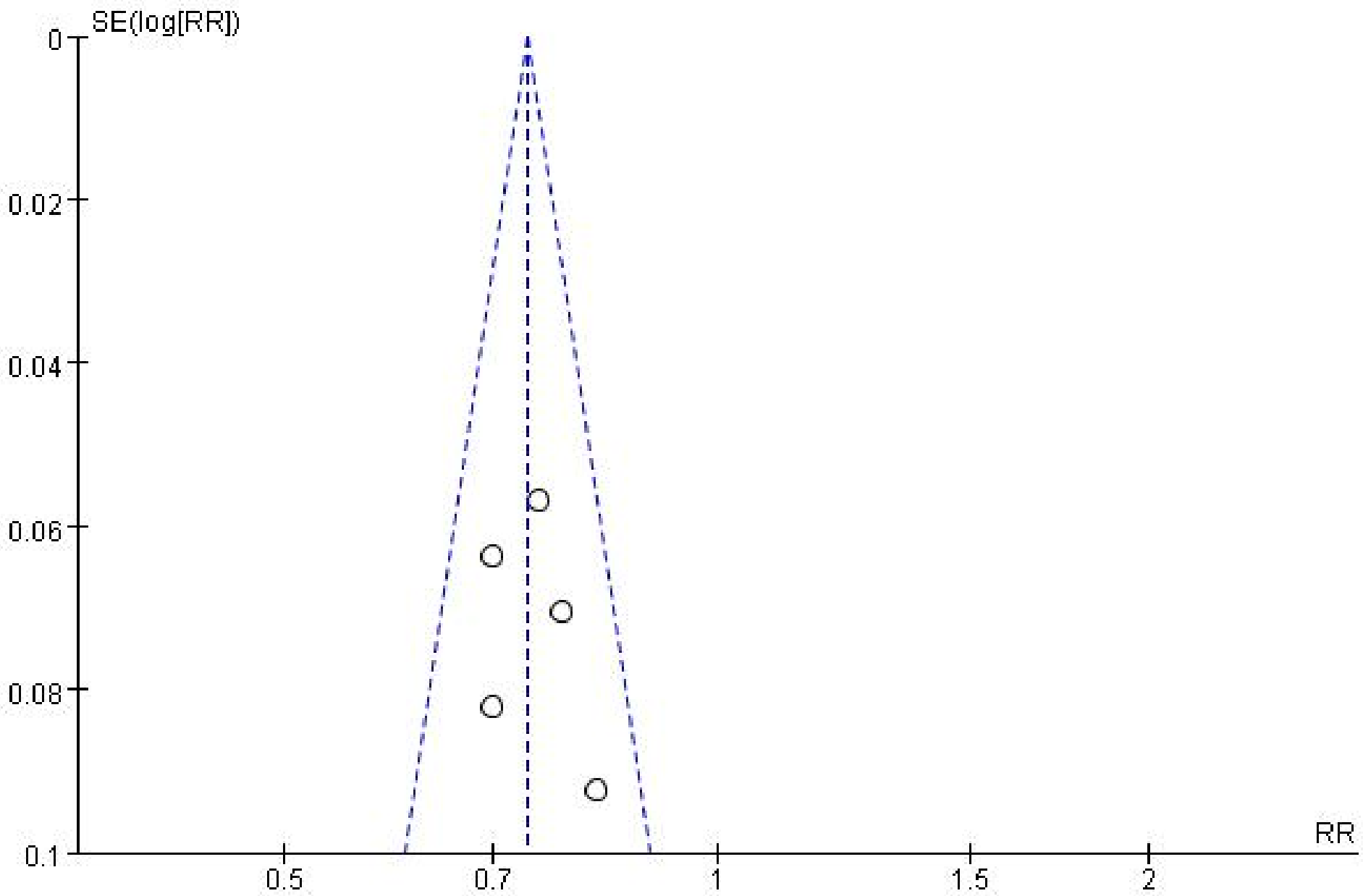

SE(log[RR])
0
0.02
0.04
0.06
0.08
0.1
RR
0.5
0.7
1
1.5
2
